# HPLC-DAD analysis of *Hyssopus Cuspidatus* Boriss extract and mensuration of its antioxygenation property

**DOI:** 10.1186/s12906-020-03016-0

**Published:** 2020-07-20

**Authors:** Lu Zhao, Zhihong Ji, Keao Li, Bo Wang, Ya Zeng, Shuge Tian

**Affiliations:** 1 College of Pharmacy, Xinjiang Medical University, Urumqi, 830011 Xinjiang China; 2 Xinjiang Qimu Medical Research Institute, Urumqi, 830002 Xinjiang China; 3 Experimental Animal Center, Xinjiang Medical University, Urumqi, 830011 Xinjiang China; 4 College of TCM, Xinjiang Medical University, Urumqi, China

**Keywords:** *Hyssopus cuspidatus Boriss*, Total polyphenol and flavonoid, HPLC-DAD, Free radical scavenging, Antioxygenation property

## Abstract

**Background:**

*Hyssopus cuspidatus* Boriss has been used as an important ethnomedicinal plant for long to eliminate phlegm, relieve cough and as well as having antibacterial, antioxygenation, and antitumor activities. In this study, the polyphenol contents, flavonoid contents, free radical scavenging assay and animal antioxygenation property assay of ethanol extract of *H. cuspidatus* were measured.

**Methods:**

This study determined the total polyphenol and flavonoid contents in *H. cuspidatus* by UV-VIS. Caffeic, ferulic, and rosmarinic acids were measured using HPLC-DAD. Free radical scavenging assay of *H. cuspidatus* was studied by colorimetric method. Animal antioxygenation property assay of *H. cuspidatus* was studied with mice by biochemical assay kits.

**Results:**

The total polyphenol and flavonoid contents of *H. cuspidatus* in 2017, 2018, 2019 were determined and the contents of *H. cuspidatus* in 2019 was the highest. In addition, rosmarinic acid was the phenolic acid with the highest content in *H. cuspidatus*. Compared with those of DPPH free radical, hydroxyl free radical, and superoxide anion free radical, the scavenging ability of *H. cuspidatus* of ABTS free radical was stronger, the average IC_50_ value was 0.0245 mg/mL. In animal antioxygenation property experiment, the model group was successfully established with decreased activities of SOD, CAT, and GSH-px and increased content of MDA. The ethanol extract of *H. cuspidatus* increased the activities of SOD, CAT, and GSH-px and reduced the content of MDA. Each group of samples and the ascorbic acid positive control group showed significant differences in the results of free radical scavenging and animal antioxygenation property experiments (*P* < 0.05).

**Conclusions:**

These results suggest that *H. cuspidatus* exerts an antioxygenation property, which can be attributed to the contents of total polyphenol and flavonoid. Given its strong antioxygenation property, *H. cuspidatus* can be used as a new natural antioxidant in food preservation and disease treatment.

## Background

Plants contain a variety of bioactive metabolites that are often used in drug development, flavoring, fragrance, and other functional products because of their antioxygenation, anti-inflammatory, and antibacterial activities [1]. *Hyssopus* has been cultivated in Europe since ancient times, and it is popular as a flavoring and medicinal plant in India. It is planted as a fragrance material in high-altitude places all over the tropics [2]. *Hyssopus cuspidatus* Boriss (*H. cuspidatus*) belongs to Labiatae, is a perennial herb or subshrub, and its dry aboveground part have been commonly used in folk medicine [3]. Although it has been cultivated in many areas of China, some wild herbs are also widely distributed in Tianshan, Altay, western Junggar, Pamir, and Kunlun Mountains [4]. The whole herb contains volatile oil, which is the most studied functional natural component at present [5]. Aside from the volatile oil, polyphenols, flavonoids, triterpenes, alkanes, and steroids are also studied as major components [6]. Modern pharmacological research shows that *H. cuspidatus* can reduce or improve airway inflammation, lower blood sugar, eliminate phlegm, and relieve cough, and has many biological activities, such as antibacterial, antioxygenation, and antitumor [7].

Free radical is the direct participants and producers of human diseases, aging and death. The imbalance of free radicals is the root of human diseases and aging [8]. Therefore, as long as the free radical in the body are maintained in the dynamic equilibrium state of generation and elimination, the diseases caused by active oxygen and lipid peroxidation can be resisted [9, 10]. Antioxidants can directly eliminate excess free radical and effectively prevent or reduce the oxidative damage caused by free radical to aid in the treatment of diseases related to oxidative damage [11, 12]. Natural antioxidants found in plants are popular because of their high efficiency and nontoxicity, and are mainly derived from plant polyphenols and flavonoids [13]. Polyphenols can inhibit the production of free radical and reactive oxygen species (ROS) in metabolism, which is related to the risk of diseases caused by oxidative stress, such as inflammation, cancer, atherosclerosis, and other chronic diseases [14]. Phenolic acid, which is the main component of polyphenols in plant food, can scavenge free radical and is related to anti-cancer and anti-inflammatory activities [15]. Flavonoids, which are strong antioxidants abundant in plants, can also effectively scavenge oxygen free radical in the body. The antioxygenation effect of flavonoids can prevent cell degeneration, aging, and cancer. Epidemiology also shows that flavonoids are potential adjuvant therapy for Alzheimer’s disease [16].

This study aims to detect the total polyphenol and flavonoid in *H. cuspidatus* samples that were collected in different years and then establish the relationship between the content and the year. High-performance liquid chromatography-diode array detector (HPLC-DAD) was used to analyze the phenolic compounds in *H. cuspidatus*. Free radical scavenging capacity and animal antioxygenation property of *H. cuspidatus* ethanol extract was tested. This study can serve as a reference to understand the antioxygenation property of *H. cuspidatus*, provide a database for the comprehensive utilization of natural antioxidants, and lay a foundation for the research of antioxygenation mechanisms.

## Methods

### Plant materials

Three wild batches of *H. cuspidatus* were obtained from Habahe County in Altay (N 48°09′81.6″, E 86°36′83.3″) in August 2017, 2018, 2019 during the flowering period. The plant is not endangered, so no permission is required to collect the samples. The plant materials were identified by Yonghe Li, a chief apothecary of the Chinese Medicine Hospital of Xinjiang. The voucher specimens (No.ZY2017081305, No.ZY2018081501, No.ZY2019082006) were deposited at the Traditional Chinese Medicine Ethnical Herbs Specimen Museum of Xinjiang Medical University.

### Chemicals and reagents

[2,2′-azino-bis-(3-ethylbenzo-thiazoline-6-sulfonic acid)] (ABTS free radical), D-galactose and ascorbic acid (VC) were received from Solarbio Science and Technology Co., Ltd. (Beijing, China). LC grade acetonitrile was supplied by Thermo Fisher Scientific Inc. (USA). Caffeic and rosmarinic acids standards were purchased from SIGMA-ALDRICH Co., Ltd. (USA). Ferulic acid was purchased from Yuanye Biotechnology Co., Ltd. (Shanghai, China). 2,2′-diphenyl-1-picrylhydrazyl (DPPH free radical) was purchased from TCI Development Co., Ltd. (Shanghai, China). The biochemical assay kits used for determination of superoxide dismutase (SOD), malondialdehyde (MDA), catalase (CAT) and glutathione peroxidase (GSH-px) were received from Elabscience Biotechnology Co., Ltd. (Wuhan, China). All the chemicals used in the study were of analytical grade.

### Extraction method

*H. cuspidatus* coarse powder was dried in an oven at 60 °C. Extraction was conducted using 2.0 g of *H. cuspidatus* coarse powder with 20 mL of 70% ethanol at 85 °C in a water bath reflux device for 1 h [17]. The samples were extracted twice and filtered. The extract was placed in a 50 mL bottle for the determination of total polyphenol content (TPC) and total flavonoid content (TFC).

In accordance with the above extraction method, 150.0 g of *H. cuspidatus* coarse powders were extracted, and the liquid extract was evaporated to dryness on a water bath. The yield of *H. cuspidatus* ethanol extract in 2019, 2018, 2017 were 10.36, 10.24, and 10.11%, respectively. Free radical scavenging assay and animal antioxygenation property assay were carried out with the residue as the sample.

One point five gram residue were dissolved in 30 mL of water and 5 mL of hydrochloric acid, heated, and then hydrolyzed in a water bath for 30 min [18]. After cooling, the samples were extracted three times with 20 mL of ethyl acetate and combined with the upper solution. The ethyl acetate extract was dried with 2.0 g of anhydrous sodium carbonate. Then, the extract was evaporated to dryness, and the residues were dissolved in 25 mL of methanol. Thus, the solutions were obtained for HPLC detection.

### TPC assay and TFC assay

The TPC assay was determined using the colorimetric method with Folin-phenol reagent [19]. In brief, 1 mL of *H. cuspidatus* extract was added to 0.5 mL of Folin-phenol reagent and 1.5 mL of sodium carbonate solution (10% w/v). After the immediate addition of 8 mL pure water, the mixture was left for 10 min in a water bath at 75 °C. Finally, an ultraviolet-visible (UV-VIS) spectrophoto meter was used to detect the absorbance at 760 nm. The standard curve of the absorbance value of gallic acid concentration solution was then determined. TPC was indicated as mg of gallic acid equivalent per g of weight of plant after drying.

The TFC assay was conducted by using the colorimetric method in line with Mellado’s report [20]. In brief, 0.5 mL of *H. cuspidatus* extract was added to 1 mL of sodium nitrite, left to stand for 6 min, added with 1 mL of 10% aluminum nitrate, and then left to stand for 6 min. Then, 10 mL of 1.0 M sodium hydroxide was added, the volume of water was fixed to 20 mL, and the solution was placed for 15 min. Finally, UV-VIS spectrometry was used to detect the absorbance at 510 nm. The standard curve of the absorbance value of rutin concentration solution was then determined. TFC was indicated as mg of rutin equivalent per g of weight of plant after drying.

### HPLC-DAD analysis of phenolic acids

HPLC-DAD system (Agilent RRLC 1200, USA) was used to separate the phenolic acids of *H. cuspidatus*. The analysis was performed with a Wondasil C18 Herb column (250 × 4.6 mm, 5 μm particle size). The mobile phase comprised 0.2% phosphoric acid in water (A) and acetonitrile (B) with a flow rate of 1 mL/min. Gradient elution was used for chromatographic analysis, and the gradient elution procedure was as follows: Phase B increased from 18 to 30% in 30 min and then to 18% in 2 min. Then, the proportionality was maintained for 6 min.

**Fig. 1:**
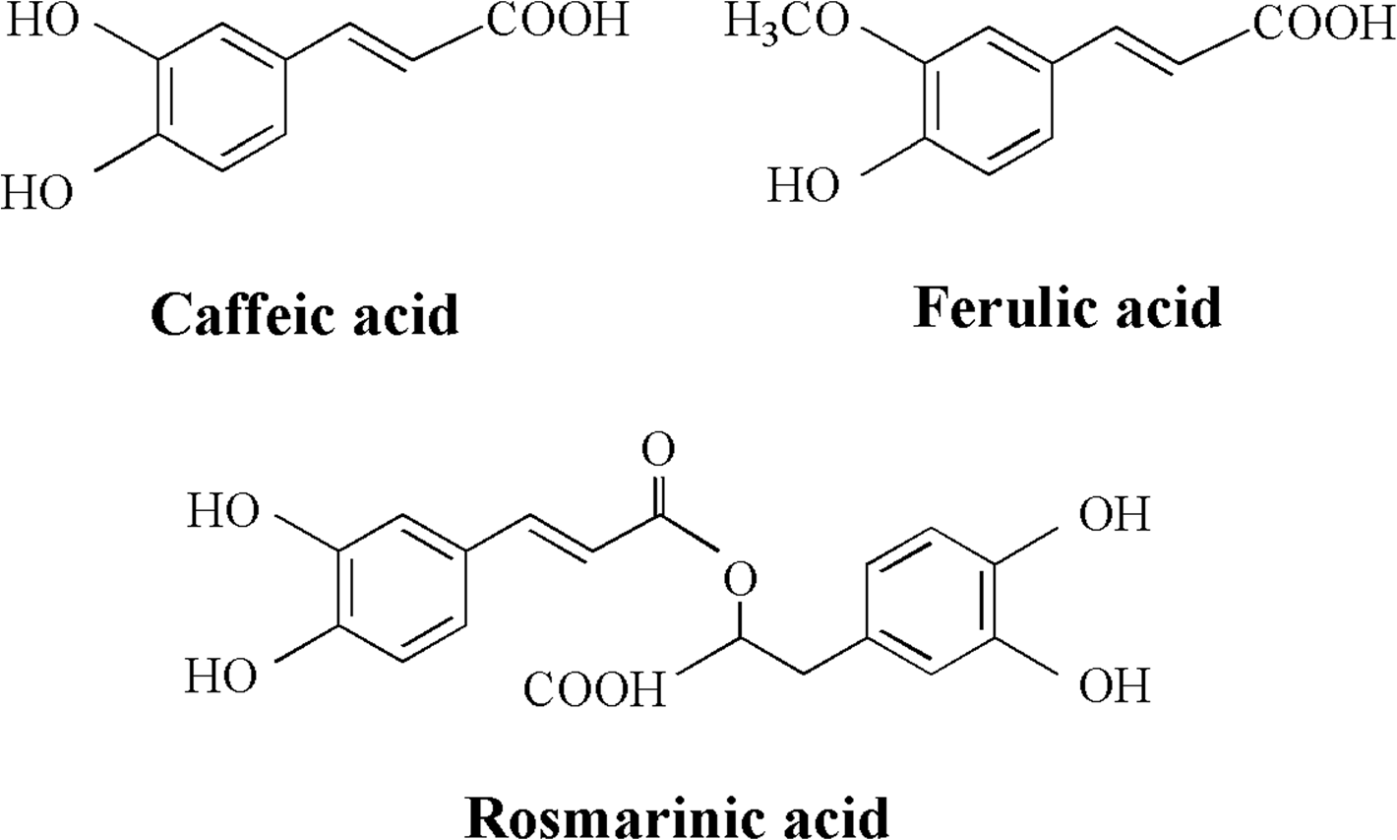
The chemical structure of caffeic, ferulic and rosmarinic acids

The extraction was injected by an auto sampler, and the injection volume was 10 μL. The detection wavelength was 325 nm, and the column temperature was 35 °C for the chromatographic analysis of phenolic acids. A standard solution containing phenol compounds, such as caffeic, rosmarinic and ferulic acids, were employed to identify and quantify the analytes. The molecular structure of the three compounds of caffeic, rosmarinic and ferulic acids in the extract of *H. cuspidatus* extract is shown in Fig. 1. Calibration curves were obtained by injecting six concentrations of mixed standard solutions, and then the sample content was obtained by calculation.

### DPPH free radical assay

The DPPH free radical scavenging assay was carried in accordance with the method described by Vlase et al. with minor modifications [21]. In brief, the *H. cuspidatus* extract was dissolved in 70% ethanol at different concentrations and mixed with 2 mL of a freshly prepared ethanol solution of DPPH free radical (100 μM). The solution was mixed vigorously and stored in darkness at room temperature for 30 min, and UV-VIS (PERSEE New century T6, China) was used to detect the absorbance at 517 nm. The positive control group was measured with VC. The results were expressed as half maximal inhibitory concentration (IC_50_), which was used to indicate the corresponding concentration of the extract when the antioxygenation free radical scavenging capacity was 50%.

### ABTS free radical assay

The ABTS free radical scavenging assay of the samples was performed in accordance with the method described by Kang et al. with slight modifications [22]. In brief, 2 mL of 10 mM potassium persulfate solution and 2 mL of 10 mM ABTS free radical solution were mixed and then stored in the dark for 12 h. Ethanol was added to the mixed solution to make its absorbance value reach 0.700 ± 0.020 as ABTS working solution at 736 nm on the UV-VIS spectra. After that, 2 mL of *H. cuspidatus* extract or ascorbic acid solution was mixed with 2 mL of ABTS working solution vigorously and stored in the dark at room temperature for 10 min. The IC_50_ values of the sample extract were calculated on the basis of the concentration and capacity of free radical scavenging curves.

### Hydroxyl free radical assay

The hydroxyl free radical assay of the samples was carried out in accordance with the method described by Lu et al. with slight corrections [23]. In brief, 0.5 mL of 7.5 mM ferrous sulfate heptahydrate, 0.5 mL of 7.5 mM salicylic acid, 1 mL of *H. cuspidatus* extract, and 0.2 mL of 30% hydrogen peroxide were mixed and left for 30 min in a water bath at 37 °C. After being cooled, the absorbance of the sample, blank, and control groups was determined at 510 nm on the UV-VIS spectrometer.

### Superoxide anion free radical assay

The superoxide anion free radical assay of the samples was performed in accordance with the method reported by Liu et al. with some revisions [24]. In brief, 4.5 mL of 50 mM Tris-Hydrochloric acid and 1 mL of *H. cuspidatus* extract were mixed and left for15 min in a water bath at 25 °C. Afterward, 0.4 mL of 5 mM pyrogallic acid was added and left for 5 min in a water bath at 25 °C. Subsequently, 0.1 mL of 8 M hydrochloric acid was added to terminate the reaction. Ultimately, the absorbance of the sample, blank, and control groups was measured instantly at 325 nm on the UV-VIS spectrometer.

### Animal antioxygenation property assay

The animal antioxygenation property assay was performed in accordance with the method reported by Wang et al. with slight revisions [25]. Male six-week-old Kunming mice weighing 20–23 g and were specific-pathogen-free were obtained from the Experimental Animal Center of Xinjiang Medical University. The mice were housed in plastic cages under ambient condition and were allowed free access to food and water. The protocol for the experiments was approved by the Ethics Committee of Experimental Animal Center of Xinjiang Medical University (IACUC20191015–01), Xinjiang, China.

After 3 days of adaptive feeding, the mice were randomly divided into six groups (*n* = 8/group): (1) normal control group (NCG): 0.9% physiological saline (10 mL/kg); (2) D-galactose model group (DMG): 0.01 mL/g D-galactose (100 mg/kg) and 0.9% physiological saline; (3) VC positive control group (VCG): VC (100 mg/kg) and 0.01 mL/g D-galactose; (4) low-concentration of *H. cuspidatus* extract group (LHG): 100 mg/kg *H. cuspidatus* extract and 0.01 mL/g D-galactose; (5) middle-concentration of *H. cuspidatus* extract group (MHG): 200 mg/kg *H. cuspidatus* extract and 0.01 mL/g D-galactose; (6) high-concentration of *H. cuspidatus* extract group (HHG): 400 mg/kg *H. cuspidatus* extract and 0.01 mL/g D-galactose. The administration above continues 3 weeks. All mice were fasted for 24 h after the last administration, and then about 1 mL of blood from the fundus vein was collected in a microcentrifuge tube. After allowing to stand, the serum was centrifuged twice at 3500×g for 10 min at 4 °C, and the activities of SOD, MDA, CAT, and GSH-px were measured in accordance with the instructions of the kit. All mice were euthanized by anesthetic. All animal experiments followed the ARRIVE guidelines.

### Statistical analysis

All samples were performed in triplicate, and the standard deviation was calculated. The data were indicated as mean ± standard deviation (SD) and documented in the respective tables or figures. The data were averaged and analyzed using Statistical Package for the Social Sciences (SPSS19.0, USA) software. First, check whether the data conforms to the normal distribution. When the data accorded with normal distribution, single-factor analysis was used to analyze the data. When the data did not conform to the distribution of positive and negative, a nonparametric test was used to analyze the data. Statistically significant difference was considered at *P* < 0.05.

## Results

### TPC and TFC determination

The results showed that the TPC and TFC of *H. cuspidatus* was 1.34–2.06% and 3.33–4.48% (Table 1). It indicated that TFC was higher than TPC in *H. cuspidatus*. There have some differences among three batches of *H. cuspidatus* about TPC and TFC. The TPC and TFC of *H. cuspidatus* were highest in 2019 (Table 1). The TPC results of statistical analysis showed that there was a significant difference between *H. cuspidatus* of 2019 and those of 2017 and 2018 (*P* < 0.05), while there was no significant difference between *H. cuspidatus* of 2017 and 2018. The TFC results of statistical analysis showed that there were significant differences between any two groups of *H. cuspidatus* (*P* < 0.05) (Table 1).

**Table 1:** The TPC and TFC in ethanol extract of *H. cuspidatus* in different years

Compounds	Regression equation	*r*^2^	Linear range (mg/ml)	Sample	Contents (mg/g)	RSD (%)
TPC	Y = 0.124X-0.009	0.9987	0.002–0.005	*H. cuspidatus*(2017)	14.198 ± 0.493	3.47
				*H. cuspidatus*(2018)	13.391 ± 0.381	2.85
				*H. cuspidatus*(2019)	20.588 ± 0.551*	2.68
TFC	Y = 11.209X-0.007	0.9999	0.010–0.060	*H. cuspidatus*(2017)	36.669 ± 0.523*	1.43
				*H. cuspidatus*(2018)	33.279 ± 0.693*	2.08
				*H. cuspidatus*(2019)	44.847 ± 1.013*	2.26

Values are presented in mean ± SD, *n* = 3. * represents the values of different *H. cuspidatus* sample group are significantly different from each other (*P* < 0.05)

### HPLC-DAD analysis

**Fig. 2:**
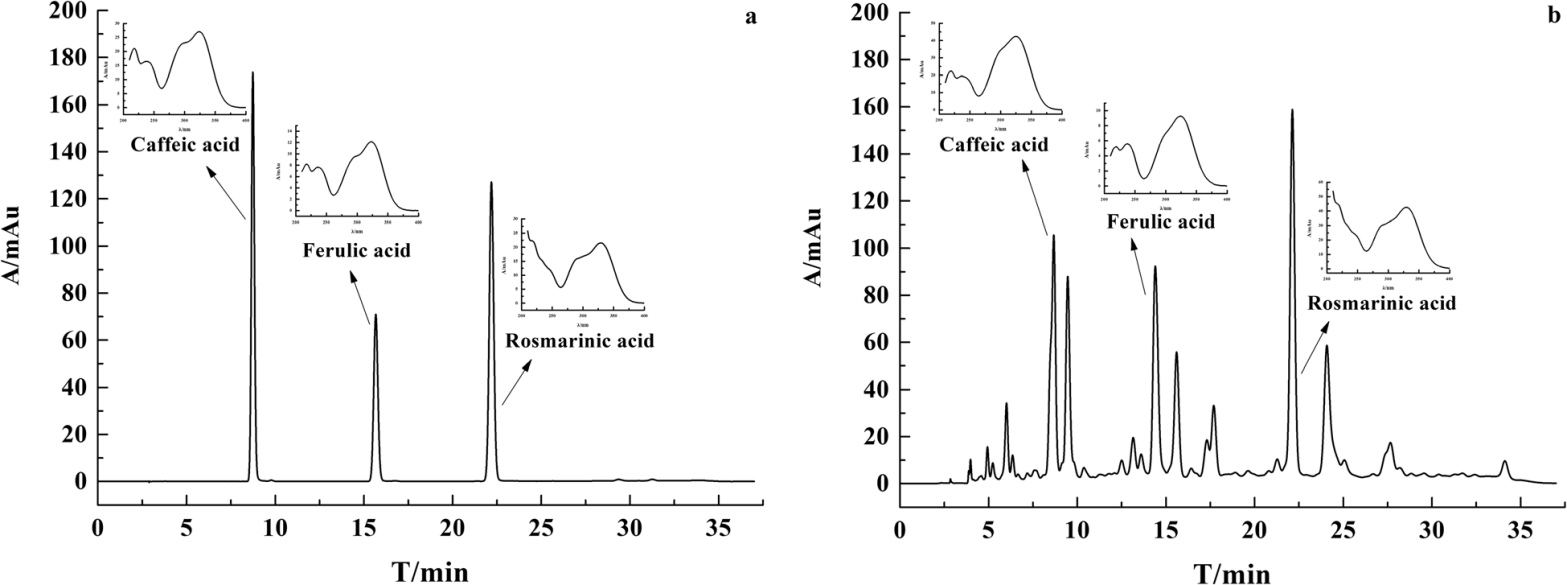
The HPLC chromatography and UV spectrum of standard and sample. (**a** represents mixed standard solution and **b** represents *H. cuspidatus* ethanol extract)

The results of HPLC-DAD showed three phenolic acids: caffeic acid (RT 8.65 min), ferulic acid (RT 15.55 min), and rosmarinic acid (RT 22.81 min) (Fig. 2). In the chromatogram of the mixed standard and sample, the peak appeared at the same relative retention time, and the whole wavelength scanning pattern of each compound peak was the same (Fig. 2). These results indicate that the sample does contain caffeic, ferulic, and rosmarinic acids. The results used for compound quantification are shown in Table 2. The content of caffeic, ferulic, and rosmarinic acids were 0.04–0.06%, 0.01–0.08 and 0.12%–0.13% (Table 3). The content of rosmarinic acid was the highest. There also have some differences among three batches of *H. cuspidatus* in HPLC-DAD analysis. The content of caffeic, ferulic, and rosmarinic acids in *H. cuspidatus* were highest in 2019 (Table 3). The statistical results showed that the content of caffeic and ferulic acids in any two groups were significantly different (*P* < 0.05). The statistical analysis showed that there was a significant difference between the rosmarinic acid content of *H. cuspidatus* of 2017 and those of 2018 and 2019 (*P* < 0.05), while there was no significant difference between *H. cuspidatus* of 2018 and 2019 (Table 3).

**Table 2:** The quantitative condition of HPLC-DAD

Compounds	Regression equation	*r*^2^	Linear range (mg/mL)	LOD (μg/mL)	LOQ (μg/mL)
caffeic acid	Y = 17,218X + 1.14	0.9996	0.040–0.242	0.259	0.746
ferulic acid	Y = 17,487X + 11.40	0.9999	0.022–0.133	0.259	0.776
rosmarinic acid	Y = 7728X + 25.16	0.9999	0.096–0.576	0.607	1.721

**Table 3:** The content of caffeic acid, ferulic acid and rosmarinic acid in *H. cuspidatus*

	Compounds	Contents (mg/g)	RSD(%)
*H. cuspidatus*(2017)	caffeic acid	0.403 ± 0.010*	2.36
	ferulic acid	0.141 ± 0.003*	2.10
	rosmarinic acid	1.238 ± 0.018*	1.44
*H. cuspidatus*(2018)	caffeic acid	0.451 ± 0.004*	0.99
	ferulic acid	0.314 ± 0.002*	0.60
	rosmarinic acid	1.296 ± 0.033	2.57
*H. cuspidatus*(2019)	caffeic acid	0.634 ± 0.004*	0.66
	ferulic acid	0.826 ± 0.003*	0.35
	rosmarinic acid	1.305 ± 0.008	0.58

Values are presented in mean ± SD, *n* = 3. * represents the values of different *H. cuspidatus* sample group are significantly different from each other (*P* < 0.05)

### Free radical scavenging assay

**Fig. 3:**
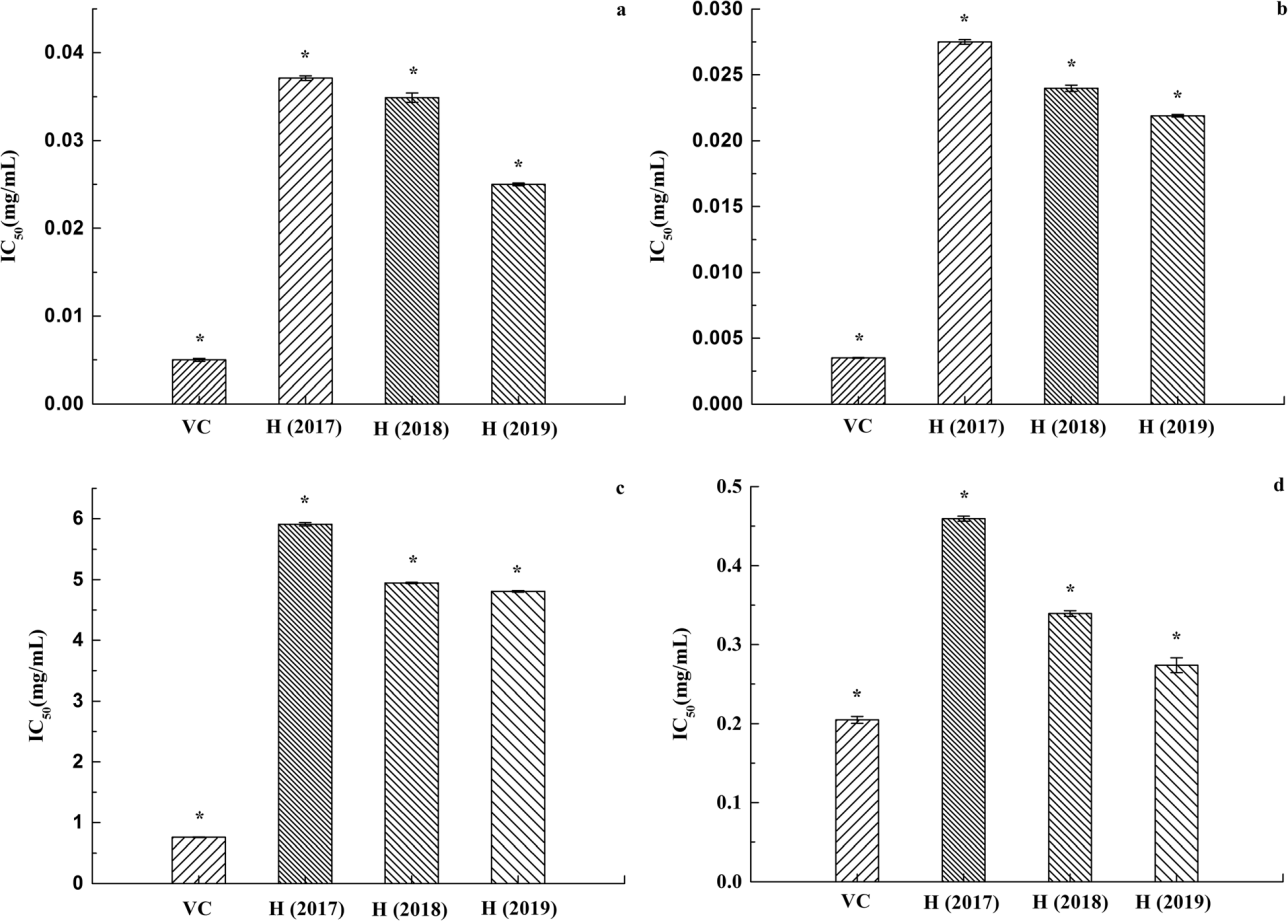
Free radical scavenging capacity of VC and ethanol extract of *H. cuspidatus* in different years. (**a** represents DPPH free radical scavenging capacity. **b** represents ABTS free radical scavenging capacity. **c** represents Hydroxyl free radical scavenging capacity. **d** represents Superoxide free radical scavenging capacity. Values were means ±SD of three replications. * represents the values of different group are significantly different from each other (*P* < 0.01))

IC_50_ is widely used as a parameter to measure antioxygenation property. The scavenging effect of the ethanol extract of *H. cuspidatus* on DPPH free radical, ABTS free radical, hydroxyl free radical and superoxide anion free radical in different years is shown in Fig. 3. The results of four free radical model assays showed that there have some differences among three batches of *H. cu*spidatus in free radical scavenging experiment. The ethanol extract of *H. cuspidatus* in 2019 had the best free radical scavenging capacity. The IC_50_ values of DPPH, ABTS, hydroxyl radical, and superoxide anion free radical were 0.0250, 0.0219, 4.8067, and 0.2738 mg/mL of *H. cuspidatus* in 2019, respectively (Fig. 3). The analysis results indicate that the antioxygenation property of the ethanol extract of *H. cuspidatus* from high to low is ABTS free radical scavenging capacity, DPPH free radical scavenging capacity, superoxide anion free radical scavenging capacity, and hydroxyl free radical scavenging capacity. SPSS single-factor analysis showed significant differences between the three ethanol extract of *H. cuspidatus* and the positive control VC (*P* < 0.01). Meanwhile, there are significant differences between any two groups of *H. cuspidatus* in free radical scavenging experiment (*P* < 0.01) (Fig. 3).

### Animal antioxygenation property assay

**Fig. 4:**
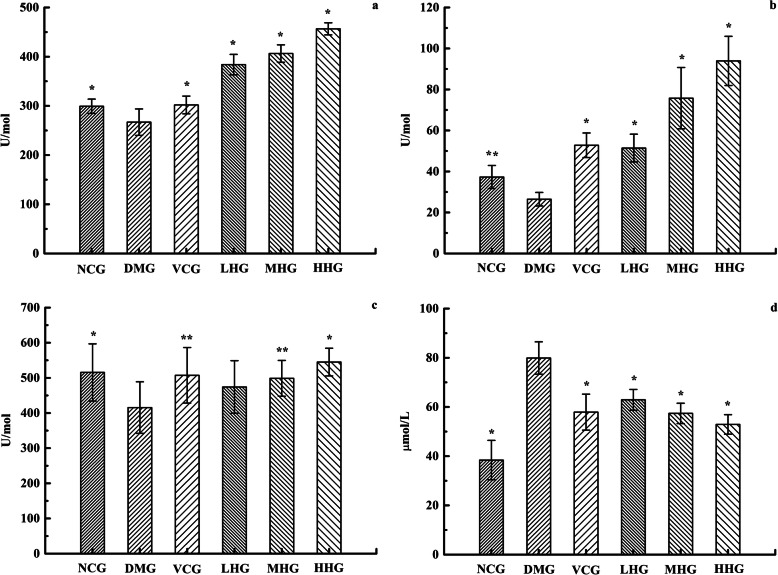
The activity of SOD, MDA, CAT and GSH-px in serum of NCG, DMG, VCG, LHG, MHG and HHG mice. (**a** represents the SOD activity. **b** represents the CAT activity. **c** represents the GSH-px activity. **d** represents the content of MDA. Values were means ±SD of eight samples in a group. * represents the values of different group are significantly different from DMG (*P* < 0.01). ** represents the values of different group are significantly different from DMG (*P* < 0.05))

The activity of SOD, MDA, CAT and GSH-px in serum of NCG, DMG, VCG, LHG, MHG and HHG mice was shown in Fig. 4. Compared with those in NCG, the activities of SOD, CAT, and GSH-px in DMG decreased while the content of MDA increased, indicating that the aging model was established successfully. For the ethanol extract of *H. cuspidatus*, at the dose of 100, 200 and 400 mg/kg body weight treatment groups significantly increased the activities of SOD, GSH-px and CAT and decreased the content of MDA as compared to DMG (*P* < 0.05) (Fig. 4). The results indicated that the *H. cuspidatus* extract in 2019 had some antioxygenation property.

All the data was analyzed by single-factor analysis. For the activity of SOD, the antioxygenation effect of LHG is much greater than that of VCG, and there was no significant difference between NCG and VCG. For CAT activity, there was no significant difference between NCG and VCG, but they had significant difference with DMG. In addition, there are significant differences between LHG, MHG and HHG of *H. cuspidatus* (*P* < 0.05) (Fig. 4). The results indicated that *H. cuspidatus* can obviously improve the activity of SOD and CAT. In view of GSH-px activity, there is significant difference between NCG, VCG, MHG, HHG and DMG, and significant difference between MHG and HHG (*P* < 0.05). However, there was no significant difference between the other groups. For the content of MDA, there was no significant difference between the VCG and sample groups, and no significant difference between LHG, MHG, and HHG. The content of MDA in VCG and sample groups was significantly higher than NCG (*P* < 0.01) (Fig. 4). The results showed that although *H. cuspidatus* had the effect of GSH-px activities and MDA content, the effect was not obvious.

## Discussion

There are differences and overlaps between flavones and polyphenols generally [26, 27]. Polyphenols and flavonoids may be the main functional components of *H. cuspidatus* to treat diseases. The determination results showed that TPC and TFC account for nearly 6% of the ethanol extract of *H. cuspidatus*. More flowers and leaves in *H. cuspidatus* appeared in 2019 because of the high freshness. In 2017 and 2018, less flowers and leaves were appeared because of storage problem. This may be one of the reasons for the higher TPC and TFC of *H. cuspidatus* in 2019. But there is still some biological activity in stem of *H. cuspidatus* in 2017 and 2018, it can be made into some compound preparation.

In HPLC-DAD analysis experiment, the separation effect of mobile phase methanol-0.1% formic acid, methanol-0.2% phosphoric acid, and acetonitrile-0.2% phosphoric acid was screened in the previous research. When the mobile phase was composed of methanol-0.1% formic acid, the separation effect was not good and many hetero peaks appeared. When the mobile phase was composed of methanol-0.2% phosphoric acid, the resolution of caffeic acid was less than 1.5. When the mobile phase was composed of acetonitrile-0.2% phosphoric acid, the target analytes peaks can be separated well. Therefore, acetonitrile-0.2% phosphoric acid aqueous solution was chosen as the mobile phase of gradient elution. The experimental results showed that the resolution of the three peaks was over 1.5, the symmetry factor was more than or equal to 0.8, and the number of theoretical plates was more than 8000. It has been reported that the ethyl acetate fraction of the areal part of *H. cuspidatus* contains caffeic acid and rosmarinic acid [1]. Consistent with the literature, their presence have been analysed by HPLC-DAD this time. The result suggests that the content of ferulic acid was directly proportional to the freshness of the samples. The highest content of ferulic acid was achieved in 2019. This result may indicate that ferulic acid existed in the flowers and leaves, whereas rosmarinic acid mainly existed in the stems. In HPLC-DAD analysis experiment, the method of sample pretreatment is complex, which may slightly affect the content of target analytes. The next step is to optimize the pretreatment method of HPLC-DAD.

Through the above experiment, the chemical constituents of *H. cuspidatus* in 2017, 2018 and 2019 were studied. The experimental results showed that there are differences between different batches of plant materials, that is, the differences between different years of the same plant material. This may be related to the soil environment, atmospheric environment, the method of plant collection and the method of storage. At the same time, the difference of chemical composition of different batches of plant materials may lead to the difference of biological activity. The results of free radical scavenging experiment showed that three batches of *H. cuspidatus* had antioxygenation property, but there also have some differences among them.

The lower the IC_50_ is, the better the antioxygenation property is; on the contrary, the higher the IC_50_ is, the worse the antioxygenation property is [28]. It has been reported that the ethyl acetate fraction of *H. cuspidatus* exhibited weak antioxygenation property, and the IC_50_ value of DPPH free radical was 26.53 ± 2.32 mg/mL [1]. In comparison, the free radical scavenging capacity of ethanol extract of *H. cuspidatus* was much stronger, the IC_50_ value of DPPH free radical was 0.0323 ± 0.0064 mg/mL, the reason may it contain more polyphenols and flavonoids. The ortho phenolic hydroxyl in the phenolic hydroxyl structure of polyphenols and flavonoids is easily oxidized to quinone structure, which consumes oxygen in the environment [29]. And it has a strong ability to capture free radical, such as ROS, therefore, polyphenols and flavonoids with strong antioxygenation property [30]. The experimental results demonstrate that ABTS free radical scavenging capacity, DPPH free radical scavenging capacity, superoxide anion free radical scavenging capacity, and hydroxyl free radical scavenging capacity is directly proportional to TFC and TPC. So the antioxygenation property of its ethanol extract of *H. cuspidatus* in 2019 is stronger than in the other years studied. The analysis results indicate that the IC_50_ value of the DPPH and ABTS free radical scavenging experiment was smaller. The types of free radicals used in different detection reaction systems, the generation methods of free radicals, the species of the base of free radical damage, and the principle of damage detection are all different [31]. As a result, the results obtained by different antioxygenation detection methods are different.

The oxidation and antioxygenation of human body should be in a relative balance [32]. The human body has a systematic antioxygenation defense mechanism that depends on three important antioxygenation enzymes: SOD, GSH-px, and CAT, which are also important indicators of ROS generation [10, 33]. In addition, MDA as an indicator of oxidative damage, its content increases with aging, and is widely used in the study of antioxygenation [34]. The animal model of subacute senescence induced by d-galactose has been widely used by scholars at home and abroad for its advantages of low cost, simple operation and stable and reliable results. D-galactose interacts with d-galactose synthase, can produce a large number of ROS, free radicals, leading to a strong oxidative stress reaction in mice, leading to cell membrane damage, and promote aging [35]. In this experiment, the activities of SOD, CAT, and GSH-px in DMG decreased while the content of MDA increased, indicating that the aging model was established successfully. The TPC and TFC in the sample of *H. cuspidatus* in 2019 are relatively large, and free radical scavenging capacity is relatively good. So, *H. cuspidatus* in 2019 was selected for animal antioxygenation property experiment. The experiment was divided into LHG, MHG and HHG, the higher the concentration of ethanol extract of *H. cuspidatus* were, the more bioactive components it contained. The activity of SOD, CAT and GSH-px is also higher, and on the contrary, the content of MDA is lower. For GSH-px activities and MDA content, there was no significant difference in LHG, MHG and HHG. It may be that GSH-px activities and MDA content are not sensitive to the change of the content of *H. cuspidatus* extract. Or for those two indicators, there is the phenomenon that the setting of high and medium and low concentrations is unreasonable.

The results of the above experiments indicated that the *H. cuspidatus* extract had antioxygenation property. But the results of free radical scavenging assay and animal antioxygenation property assay are somewhat inconsistent. The free radical scavenging assay is an in vitro experiment, whereas the animal antioxygenation property assay is an in vivo experiment. This may be due to the difference of the factors influencing in vivo and in vitro experiment*.* Free radical scavenging assay results showed that the antioxygenation property of positive control VC was stronger than that of *H. cuspidatus* extract. However, in animal antioxygenation property assay, the antioxygenation property of *H. cuspidatus* extract was stronger than VC. In vitro assay revealed that the main factors affecting antioxygenation property are the inherent characteristics of the body and the extraction process itself [36]. Nevertheless, in vivo assay may be affected by other factors, such as digestibility, bioavailability, and metabolism [37]. In vitro assay, in vivo biological effects, for instance, antioxygenation enzyme activity, oxidative metabolism pathway, and the activation or inhibition of antioxygenation and enzyme gene expression, are generally ignored [38]. Consequently, in vivo assay is suitable to assess the antioxygenation potential of compounds.

## Conclusions

In the research, the chemical composition and antioxygenation property of *H. cuspidatus* were studied. The results showed 2% total polyphenol and 4% total flavonoid in *H. cuspidatus*. The contents of caffeic, ferulic and rosmarinic acids were analyzed by HPLC-DAD, among which rosmarinic acid obtained the highest. The results of free radical scavenging assay showed that the ethanol extract of *H. cuspidatus* exerted significant scavenging effects on ABTS, DPPH, hydroxyl, and superoxide anion radical. The results of animal antioxygenation property experiment showed that the ethanol extract of *H. cuspidatus* can significantly enhance the activities of SOD, CAT, and GSH-px in mouse serum and reduce the content of MDA. In addition, the antioxygenation property of the ethanol extract of *H. cuspidatus* may be related to TPC and TFC. The higher the TPC and TFC are, the better the antioxygenation property is. Given its strong antioxygenation property, *H. cuspidatus* can be used as a new natural antioxidant in food preservation and disease treatment.

## Supplementary information

**Additional file 1.** : Ethical proof of animal experiment (PDF).

**Additional file 2.** : Fig. A The HPLC chromatography with mobile phase was composed of methanol-0.1% formic acid.

**Additional file 3.** : Fig. B The HPLC chromatography with mobile phase was composed of methanol-0.2% phosphoric acid.

## Data Availability

Data sharing is not applicable to this article as no datasets were generated or analysed during the current study.

## Abbreviations

*H. cuspidatus*: *Hyssopus cuspidatus* Boriss
ROS: Reactive oxygen species
HPLC-DAD: High-performance liquid chromatography-diode array detector
ABTS free radical: 2,2'-azino-bis-(3-ethylbenzo-thiazoline-6-sulfonic acid)
DPPH free radical: 2,2'-diphenyl-1-picrylhydrazyl
VC: Ascorbic acid
SOD: Superoxide dismutase
MDA: Malondialdehyde
CAT: Catalase
GSH-px: Glutathione peroxidase
TPC: Total polyphenol content
TFC: Total flavonoid content
UV-VIS: Ultraviolet-visible
IC_50_: Half maximal inhibitory concentration
NCG: Normal control group
DMG: D-galactose model group
VCG: VC positive control group
LHG: Low-concentration of *H. cuspidatus* extract
MHG: Middle-concentration of *H. cuspidatus* extract
HHG: High-concentration of *H. cuspidatus* extract
SD: Standard deviation
SPSS: Statistical Package for the Social Sciences
