# Supplementary material for: HPLC-DAD analysis of Hyssopus Cuspidatus Boriss extract and mensuration of its antioxygenation property
Source: BMC Complement Med Ther. 2020 Jul 20;20:228. doi: 10.1186/s12906-020-03016-0 (PMC7370466; doi:10.1186/s12906-020-03016-0)

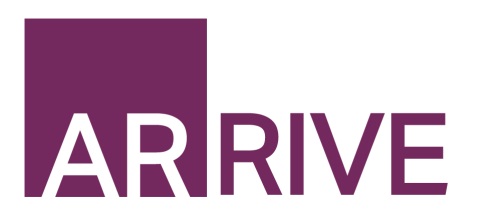


The ARRIVE Guidelines Checklist

Animal Research: Reporting In Vivo Experiments

Carol Kilkenny^1^, William J Browne^2^, Innes C Cuthill^3^, Michael Emerson^4^ and Douglas G Altman^5^

*^1^The National Centre for the Replacement, Refinement and Reduction of Animals in Research, London, UK, ^2^School of Veterinary Science, University of Bristol, Bristol, UK, ^3^School of Biological Sciences, University of Bristol, Bristol, UK, ^4^National Heart and Lung Institute, Imperial College London, UK, ^5^Centre for Statistics in Medicine, University of Oxford, Oxford, UK.*

|  | | ITEM | RECOMMENDATION | Section/ Paragraph |
| --- | --- | --- | --- | --- |
| 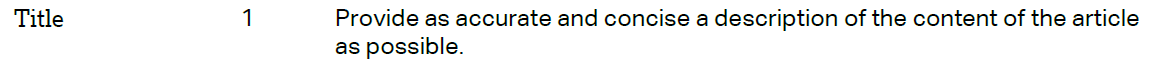 | | | Title |  |
| 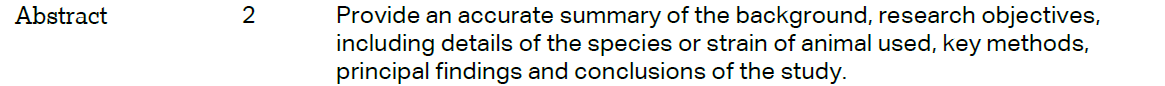 | | | Abstract |  |
| INTRODUCTION | | |  |  |
| 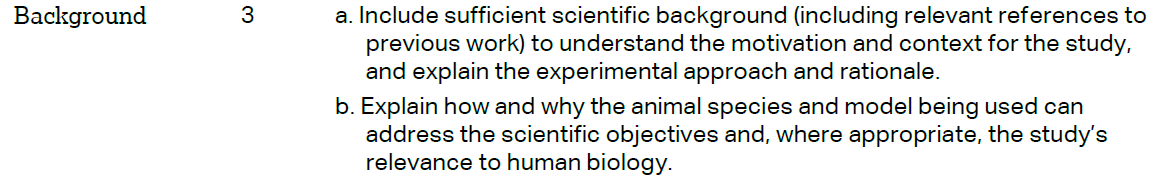 | | | Background/ Paragraphs 1-2 |  |
| 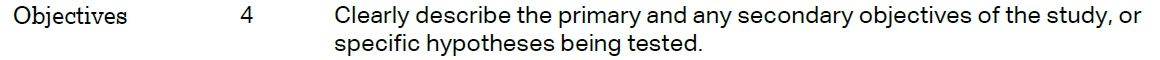 | | | Background/ Paragraph 3 |  |
| METHODS | | |  |  |
| 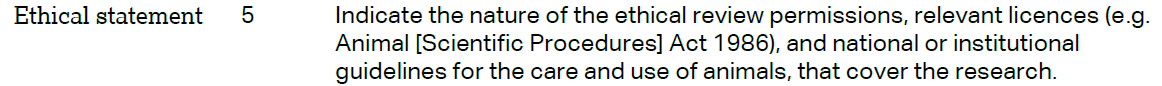 | | | Declarations/ Paragraph 1 |  |
| 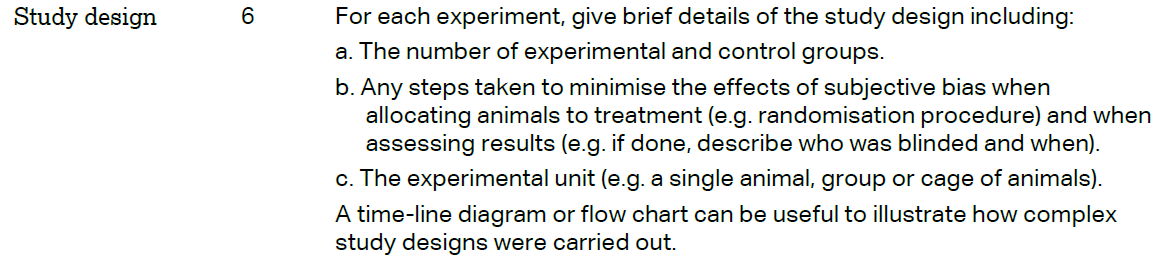 | | | Methods/ Paragraph 3-14  N/A |  |
| 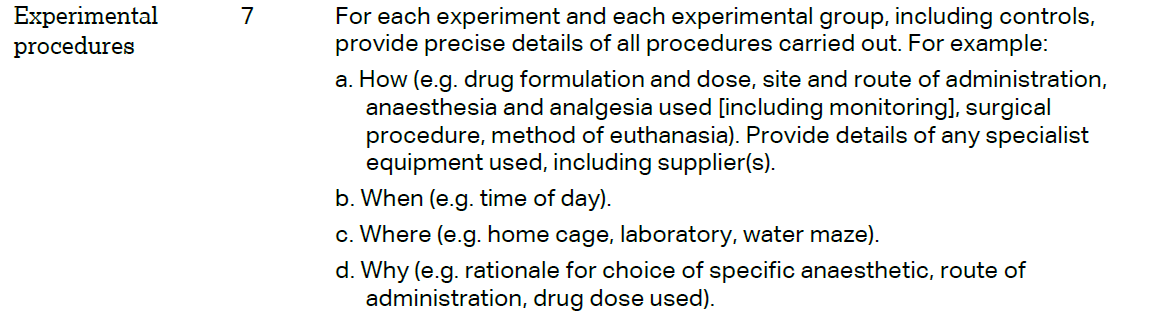 | | | Methods/ Paragraph 14 |  |
| 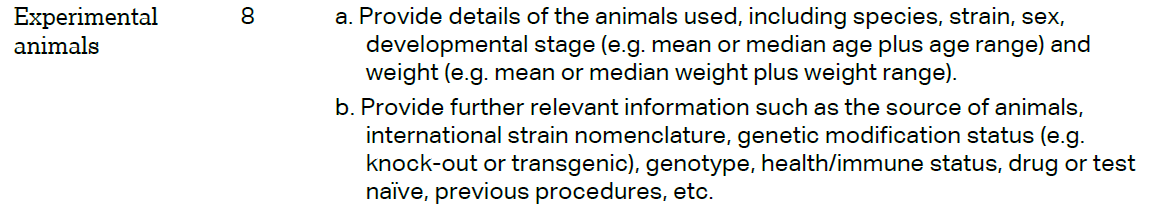 | | | Methods/ Paragraph 14 |  |

The ARRIVE guidelines. Originally published in *PLoS Biology*, June 2010^1^

| 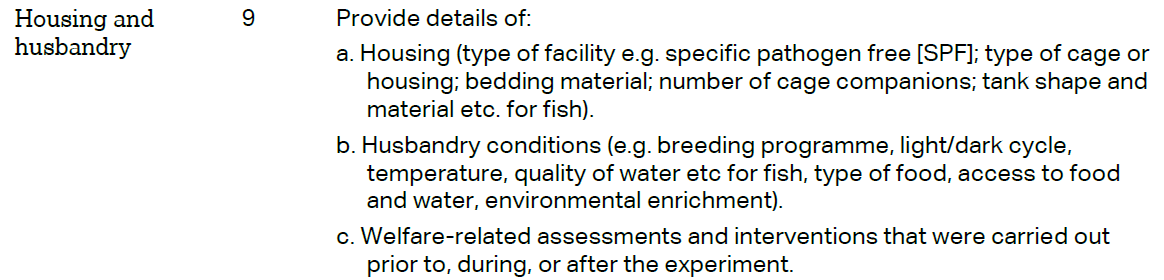 | Methods/ Paragraph 14 | |
| --- | --- | --- |
| 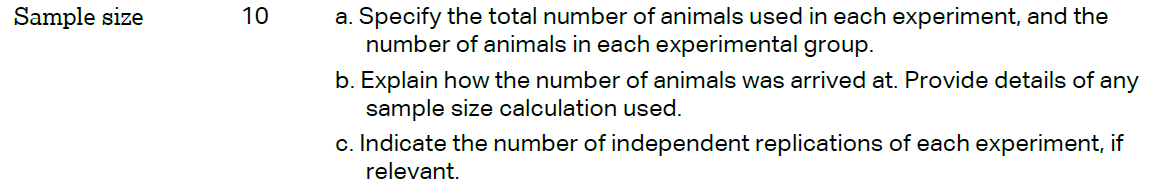 | Methods/ Paragraph 14 | |
| 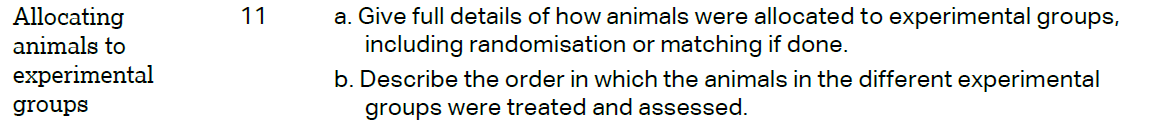 | Methods/ Paragraph 14 | |
| 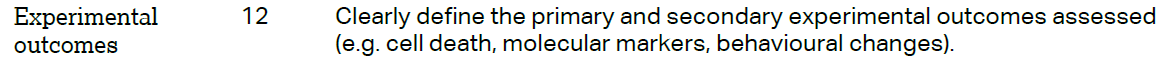 | Methods/ Paragraph14 | |
| 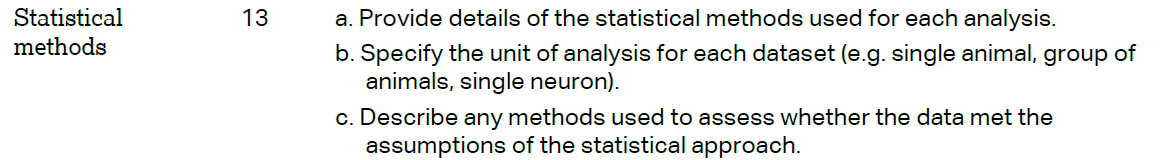 | Methods/ Paragraph 14&15  Results/Paragraph 1-5 | |
| RESULTS |  | |
| 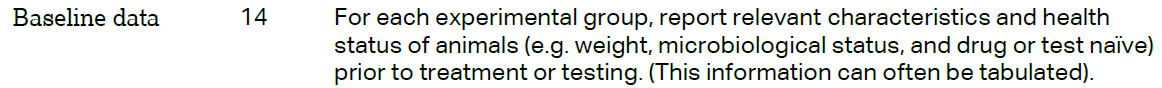 | N/A | |
| 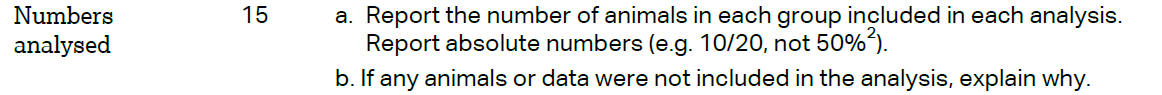 | Methods/ Paragraph 14 | |
| 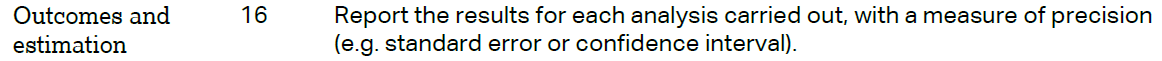 | Results/Fig. 3&4, Table 1&3 | |
| 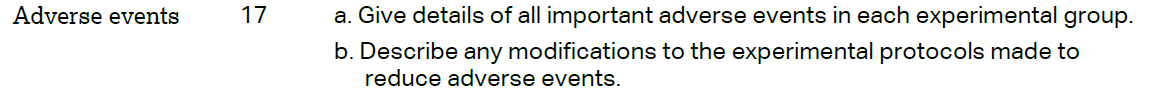 | N/A | |
| DISCUSSION |  | |
| 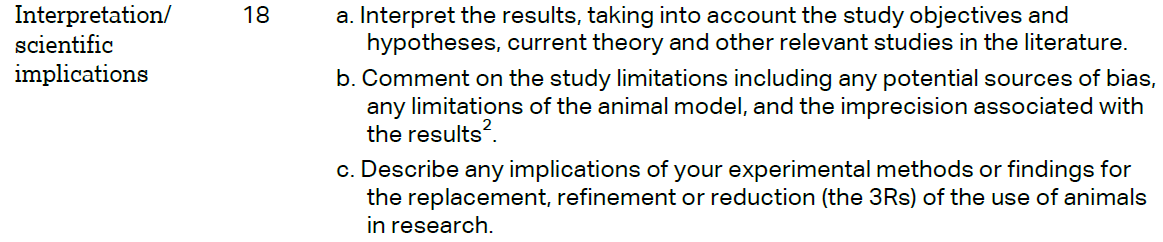 | Discussion/ Paragraphs 1-6 | |
| 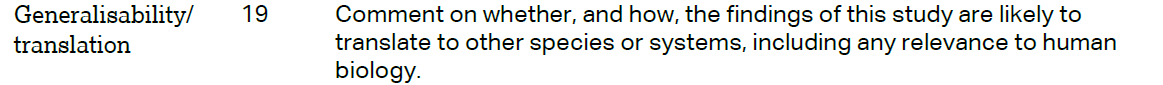 | Discussion/ Paragraphs 6 | |
| 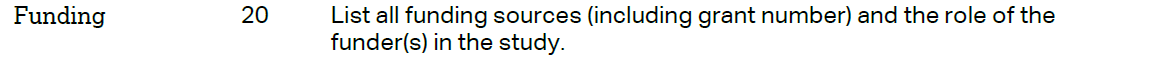 | | Funding |


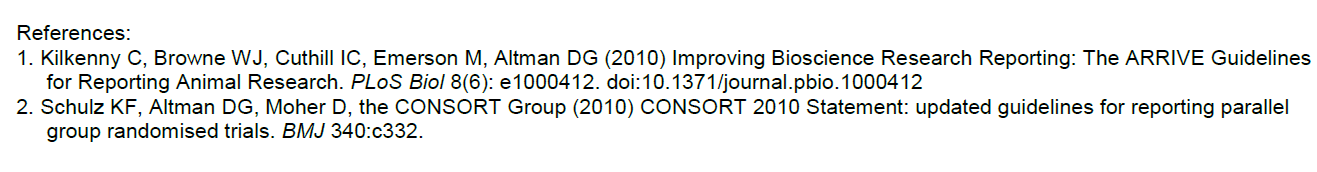

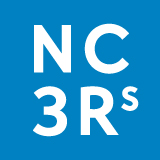

Supplement: Supplementary file 1 — Additional file 1. : Ethical proof of animal experiment (PDF). [file 12906_2020_3016_MOESM1_ESM.docx]
